# Artificial intelligence demonstrates potential to enhance orthopaedic imaging across multiple modalities: A systematic review

**DOI:** 10.1002/jeo2.70259

**Published:** 2025-05-07

**Authors:** Umile Giuseppe Longo, Alberto Lalli, Guido Nicodemi, Matteo Giuseppe Pisani, Alessandro De Sire, Pieter D'Hooghe, Ara Nazarian, Jacob F. Oeding, Balint Zsidai, Kristian Samuelsson

**Affiliations:** ^1^ Fondazione Policlinico Universitario Campus Bio‐Medico Roma Italy; ^2^ Department of Medicine and Surgery Research Unit of Orthopaedic and Trauma Surgery, Università Campus Bio‐Medico di Roma Roma Italy; ^3^ Department of Medical and Surgical Sciences University of Catanzaro “Magna Grecia” Catanzaro Italy; ^4^ Department of Orthopaedic Surgery and Sportsmedicine Aspetar Hospital Doha Qatar; ^5^ Carl J. Shapiro Department of Orthopaedic Surgery Musculoskeletal Translational Innovation Initiative, Beth Israel Deaconess Medical Center, Harvard Medical School Boston Massachusetts USA; ^6^ School of Medicine, Mayo Clinic Alix School of Medicine Rochester Minnesota USA; ^7^ Department of Orthopaedics Institute of Clinical Sciences, The Sahlgrenska Academy University of Gothenburg Gothenburg Sweden

**Keywords:** artificial intelligence (AI), imaging, multimodality

## Abstract

**Purpose:**

While several artificial intelligence (AI)‐assisted medical imaging applications are reported in the recent orthopaedic literature, comparison of the clinical efficacy and utility of these applications is currently lacking. The aim of this systematic review is to evaluate the effectiveness and reliability of AI applications in orthopaedic imaging, focusing on their impact on diagnostic accuracy, image segmentation and operational efficiency across various imaging modalities.

**Methods:**

Based on the PRISMA guidelines, a comprehensive literature search of PubMed, Cochrane and Scopus databases was performed, using combinations of keywords and MeSH descriptors ('AI', 'ML', 'deep learning', 'orthopaedic surgery' and 'imaging') from inception to March 2024. Included were studies published between September 2018 and February 2024, which evaluated machine learning (ML) model effectiveness in improving orthopaedic imaging. Studies with insufficient data regarding the output variable used to assess the reliability of the ML model, those applying deterministic algorithms, unrelated topics, protocol studies, and other systematic reviews were excluded from the final synthesis. The Joanna Briggs Institute (JBI) Critical Appraisal tool and the Risk Of Bias In Non‐randomised Studies‐of Interventions (ROBINS‐I) tool were applied for the assessment of bias among the included studies.

**Results:**

The 53 included studies reported the use of 11.990.643 images from several diagnostic instruments. A total of 39 studies reported details in terms of the Dice Similarity Coefficient (DSC), while both accuracy and sensitivity were documented across 15 studies. Precision was reported by 14, specificity by nine, and the F1 score by four of the included studies. Three studies applied the area under the curve (AUC) method to evaluate ML model performance. Among the studies included in the final synthesis, Convolutional Neural Networks (CNN) emerged as the most frequently applied category of ML models, present in 17 studies (32%).

**Conclusion:**

The systematic review highlights the diverse application of AI in orthopaedic imaging, demonstrating the capability of various machine learning models in accurately segmenting and analysing orthopaedic images. The results indicate that AI models achieve high performance metrics across different imaging modalities. However, the current body of literature lacks comprehensive statistical analysis and randomized controlled trials, underscoring the need for further research to validate these findings in clinical settings.

**Level of evidence:**

Systematic Review; Level of evidence IV.

**Abbreviations**
3D dGEMRIC: 3D delayed gadolinium‐enhanced MRI of cartilage
3D_WATS: three‐dimensional water‐selective
3D‐UNet: 3D U‐shaped architecture network
ACL: anterior cruciate ligament
AI: artificial intelligence
AP: anteroposterior
cGAN: conditional generative adversarial network
CNN: convolutional neural network
CRF: conditional random field
D2FC‐DN: deeply supervised dilated fully convolutional‐DenseNet
DESS: double‐echo steady‐state
DL: deep learning
DR: digital radiography
DSC: dice similarity coefficient
FAI: femoroacetabular impingement
FCN: full convolutional neural networks
FCNN‐4s: fully convolutional neural network
FHC: femoral head coverage
GAN: generative adversarial networks
HR‐pQCT: high‐resolution peripheral quantitative computed tomography
IFF: intertrochanteric femoral fracture
IPLF: integrated‐on‐positive‐loss function
IVD: intervertebral disk
IVDD: intervertebral disc degeneration
LIVF: lumbar intervertebral foramen
ML: machine learning
MRI: magnetic resonance imaging
Msge: multiscale geometric information embedded
nnU‐Net: not new network
OA: osteoarthritis
PCC: prospective case–control
PCNN: pulse coupled neural network
PCS: prospective case‐series
RCC: retrospective case–control
R‐CNN: regions with convolutional neural network
RCS: retrospective case‐series
ROM: range of motion
SMART‐CA: segmentation model adopting a pRe‐trained classification architecture
TKA: total knee arthroplasty
UNETR: U‐net transformer
US: ultrasound
VBS: vertebral bone structures
VDVM: vertebral segment matching

## INTRODUCTION

Artificial intelligence (AI) has the potential to enhance medical diagnostics, clinical decision‐making and outcome prediction [66], through the pattern‐learning and accurate predictive capabilities of machine‐learning (ML) models. Training ML models on several imaging modalities, including radiography, magnetic resonance imaging (MRI), computed tomography (CT) and ultrasonography (US), may further enhance the diagnostic process.

Recent explorations into AI applications in diagnostic imaging have shown promising outcomes in enhancing image quality, and accelerating processing times, thus potentially increasing the overall diagnostic accuracy [32]. In the orthopaedic field, AI applications not only enhance image segmentation, but also improves 3D model generation and surgical planning, using technologies like convolutional neural networks (CNNs) and generative adversarial network (GAN).

A study by Krogue et al. [27] demonstrated that deep learning (DL) algorithms could accurately identify hip fractures and provide functional subclassifications, enhancing diagnostic precision and aiding in treatment planning. Similarly, Jones et al. [17] found that a DL system for fracture detection in musculoskeletal radiographs could match or exceed the performance of human radiologists.

Additionally, AI has enhanced the segmentation and analysis of MRI images, such as in the automatic segmentation of rotator cuff muscles, aiding in the diagnosis and treatment planning of rotator cuff tears [38].

AI also supports real‐time, intraoperative analysis, advancing less invasive procedures and evolving orthopaedic diagnostics and treatments [1, 2, 3, 4, 5, 6, 7, 9, 10, 12, 13, 15, 16, 18, 20, 21, 22, 23, 24, 25, 26, 28, 29, 31, 33, 34, 35, 36, 39, 42, 43, 44, 45, 46, 47, 49, 50, 51, 52, 53, 54, 55, 56, 57, 58, 59, 60, 61, 62, 63, 64, 65, 67]. However, despite this progress, the quality of studies and systematic analyses evaluating these new AI tools varies, and a structured framework for assessing their effectiveness and reliability is often lacking. Moreover, while AI offers substantial benefits, integrating these systems into clinical workflows must ensure the interpretability of AI decisions, managing data privacy concerns, and overcoming institutional resistance to technological change. Furthermore, ethical considerations remain at the forefront of discussions about AI in healthcare, particularly regarding algorithmic bias [19] and the concerns about using AI as a tool for expert radiologists versus as a complete substitute for professionals.

This systematic review aims to elucidate the current advancements and applications of ML in diagnostic accuracy and operational efficiency in different medical imaging modalities, evaluating the reported outcomes.

Our goal is to provide a thorough analysis of the benefits, challenges, and future directions of AI applications in imaging, contributing to a deeper understanding and a more effective implementation of these technologies in clinical practice.

## MATERIALS AND METHODS

### Eligibility criteria

A systematic review of literature was carried out in March 2024. Full‐text articles written in English or Italian were included and encompassed both prospective and retrospective studies, as well as case‐series and case‐control studies. The review focused on adult populations with participants being >18 years old. The studies selected for inclusion were those that investigated the capability of AI to improve the quality of diagnostic images and to ease the analysis and the segmentation of the body regions. Review articles, non‐clinical studies, editorials, letter to editor, conference commentary, protocol studies, and all articles that used deterministic algorithms or AI to predict the cost of healthcare, were excluded. Articles eligible for inclusion assessed at least one of the following outcomes: dice similarity coefficient (DSC), area under the curve (AUC), accuracy, F1 Score, precision, sensitivity and specificity.

### Information sources

**Figure 1:**
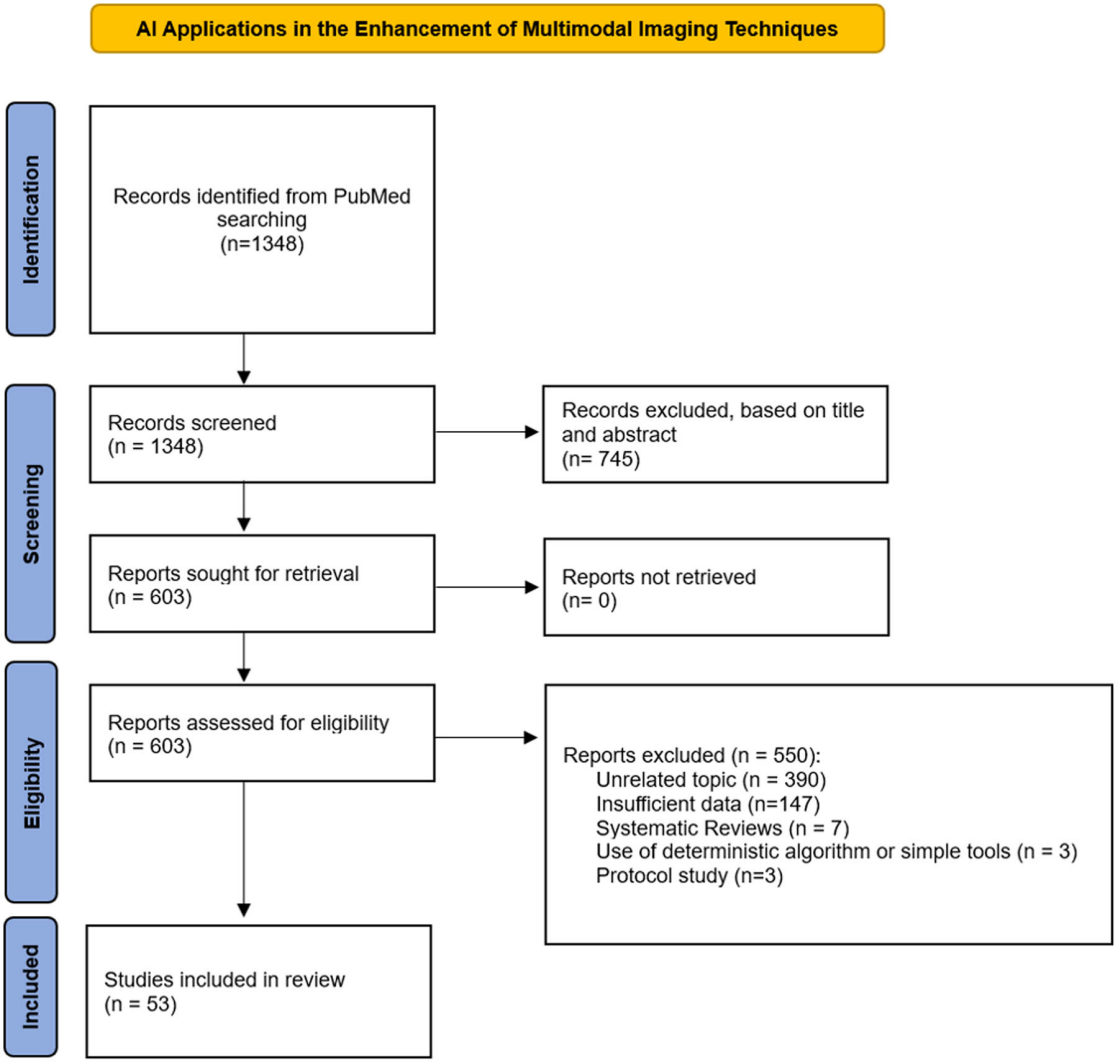
Selection flow diagram according to the PRISMA 2020 statement. From: Page MJ, McKenzie JE, Bossuyt PM, Boutron I, Hoffmann TC, Mulrow CD, et al. The PRISMA 2020 statement: an updated guideline for reporting systematic reviews. BMJ 2021;372:n71. 10.1136/bmj.n71. For more information, visit: http://www.prisma-statement.or.

A systematic literature search of the following bibliographic databases was completed: the US National Library of Medicine (PubMed/MEDLINE), SCOPUS, Cochrane Database of Systematic Reviews and EMBASE. The search was performed following The Preferred Reporting Items for Systematic Reviews and Meta‐analysis (PRISMA) guidelines (Figure 1) [37].

### Search strategy

The search strategies used a combination of Medical Subject Heading (MeSH) terms and “title/abstract” search. Similar search strategies were used across all databases, with minor differences when necessary, due to specific database configurations: '((((artificial intelligence) OR (machine learning) OR (deep learning))) AND ((orthopedic surgery)) AND (imaging))'.

### Selection process

Title and abstract screening were performed first, by two independent reviewers (M.G.P., G.N.). The same reviewers performed full text screening of the selected articles to verify if they met the eligibility criteria. Differences or disagreements at any stage were resolved with consultation of a third reviewer (A.L.).

### Data collection process

After the selection of eligible studies, data was extracted and divided into three tables, reporting (Table 1) the name of the first author, year of publication, study design, level of evidence, aim, sample size; in Table 2, the joint or body region involved, type of imaging technique and AI model; in Tables 3, 4, 5, 6, 7, 8, 9, the outcomes of the ML application. For studies with incomplete data or with data that was not directly detectable, an initial attempt was made to contact the corresponding author for feedback. In case of non‐response or inability to provide additional data, other reviews were checked to verify the presence of the data of interest published in other systematic reviews.

**Table 1:** Demographics.

First author	Year	Joint/body region	Imaging	AI
Ahn G.	2022	Knee joint	X‐Ray	GAN
Alsinan A.	2019	Radius, femur, tibia	US	CNN
Alsinan A.	2020	Radius, femur, tibia, spine	US	GAN
Burton W.	2020	Knee joint	MRI	CNN
Chaudhari A.S.	2020	Knee joint	3D DESS MRI	CNN
Chen T.	2022	Lumbar spine	3D MRI T2	3D U‐Net
El‐Hariri H.	2021	Hip joint	US	3D CNN
Flannery S.W.	2021	Knee joint	MRI T2	2D U‐Net
Flannery S.W.	2022	Knee joint	MRI T3	2D U‐Net
Hess H.	2023	Shoulder joint	MRI T1	nnU‐Net
Hwang D.	2022	Knee joint	X‐Ray	U‐Net; XGBoost
Jecklin S.	2022	Lumbar spine	X‐Ray	X23D algorithm
Jeon U.	2021	Knee joint	MRI	2D U‐Net
Kashua S.	2023	Knee joint	MRI T1	2D CNN
Kim H.	2022	Shoulder joint	MRI T2	nnU‐Net
Kim H.	2023	Tibia, Fibula	2D/3D CT	DeepLab v3+
Kim HS.	2024	Hip joint, thigh muscles	CT	UNETR
Kim S	2018	Spine	MRI	BSU‐Net
Kim‐Wang S.Y.	2023	Knee joint	MRI	U‐Net
Kok J.	2023	Spine	MRI	nnU‐Net
Kokita S	2020	Abdominal cavity, bones, muscle, subcutaneous tissue and thoracic cavit	CT	3D U‐Net
Kulsen C.P.S	2023	Knee joint	MRI	DenseVNet
Kuok C.P	2020	Fingers	US	D2FC‐DN
Kwolek K	2023	Knee joint	X‐Ray	U‐Net; Yolo
Lee K.	2021	Shoulder joint	US	SMART‐CA
Lee S.	2024	Wrist joint	X‐Ray	U‐Net
Lee SH.	2023	Shoulder joint	MRI	3D U‐Net; CNN
Li X.	2023	Hip joint	MRI T2	MsgeCNN
Luan K.	2020	Tibia, radius	US	BoneNet
Marsilio L.	2023	Tibia, femur	CT	CEL‐Unet
Masse‐Gignac N.	2023	Spine	MRI T2	CNN
Medina G	2021	Shoulder joint	MRI T1	CNN
Mu X.	2021	Shoulder joint	MRI	U‐Net; Alex Net
Nishiyama D.	2021	Gluteus medius	CT	GAN
Olmez E.	2020	Knee joint	MRI	R‐CNN
Riem L.	2023	Shoulder joint	MRI T1	3D U‐Net
Sàeenz‐Gamboa J.	2023	Lumbar spine	MRI	U‐net
Schacky C.	2021	Bone tumors	X‐Ray	DL
Schmaranzer F.	2019	Hip joint	MRI	3‐D dGEMRIC
Sezer A.	2023	Hip joint	US	Mask R‐CNN
Shen H.	2021	Lumbar spine	MRI T2W	Spine Explorer (Tulong)
Walle M.	2022	Radius	HR‐pQCT images	CNN
Wang D.	2022	Femur	CT	V‐Net
Wang G.	2020	Humerus, shoulder joint	MRI	PCNN, FCN
Wong V.	2023	Scapula	MRI	CNN
Wu S.	2023	Femur, tibia, fibula, radius, humerus	CT	FCNN‐4s,CRF
Yang L.	2022	Femur	CT	DenseUnet, MaskR‐CNN
Yang M.	2022	Knee joint	MRI	cGAN
Zang Q.	2023	Knee joint	MRI	nnU‐Net
Zeng G. Degonda C.	2021	Femur, hip joint	CT, MRI	CNN
Zeng G. Schmaranzer F.	2021	Femur, hip joint	MRI	3D LP‐U‐net
Zhang R.	2023	Spine	DR, X‐ray	VDVM
Zheng H.	2022	Lumbar intervertebral disc	MRI	BianqueNet

Abbreviations: 3D dGEMRIC, 3D Delayed gadolinium‐enhanced MRI of cartilage; 3D‐UNet, 3D U‐shaped architecture network; AI, artificial Intelligence; cGAN, conditional generative adversarial network; CNN, convolutional neural network; CT, computed tomography; DR, digital radiography; FCN, full convolutional neural networks; FCNN‐4s, fully convolutional neural network; MRI, magnetic resonance imaging; nnU‐Net, not new network; PCNN, pulse coupled neural network; SMART‐CA, Segmentation Model Adopting a pRe‐trained Classification Architecture; VDVM, vertebral segment matching.

**Table 2:** AI Models.

First author	Year	Models	DSC
Alsinan A.	2020	Single model	0.67
Burton W.	2020	Single model	0.989
Chen T.	2022	VBS and IVD	0.918
El‐Hariri H.	2021	Single model	0.85
Flannery S.W.	2021	Single model	0.84
Flannery S.W.	2022	Single model	0.80
Hess H.	2023	Single model	0.91
Jeon U.	2021	Medial meniscus	0.851 ± 0.127
		Lateral meniscus	0.843 ± 0.126
Kim H.	2022	2D	0.810 ± 0.063
		3D	0.830 ± 0.073
Kim HS.	2024	Single model	0.84
Kim S	2018	Single model	0.894
Kim‐Wang S.Y.	2023	Validation set	0.985
		Testing set	0.984
Kok J.	2023	Healthy	0.95
		Scoliotic spine	0.93
Kokita S	2020	Average	0.955
Kulsen C.P.S	2023	Average	0.967 ± 0.040
Kuok C.P	2020	Chuang's dataset‐Tendon	0.93
		Chuang's dataset‐Synovial sheath	0.91
		MB dataset‐Tendon	0.92 ± 0.04
		MB dataset‐Synovial sheath	0.95 ± 0.03
Lee K.	2021	Single model	0.736
Lee SH.	2023	Single model	0.943
Li X.	2023	Single model	0.933 ± 0.955
Luan K.	2020	Test set 1	0.93
		Test set 2	0.912
Marsilio L.	2023	Single model	0,991
Masse‐Gignac N.	2023	Single model	0,953
Medina G	2021	Internal test dataset	0.994
		External test dataset	0.989
Mu X.	2021	Alex Net	0.910 ± 0.02
		VGG16	0.790 ± 0.02
		Inception 3	0.820 ± 0.02
		ResNet	0.800 ± 0.02
Nishiyama D.	2021	Single model	0.748
Olmez E.	2020	Single model	0.888 ± 0.075
Riem L.	2023	RCT‐One‐stage model	0.93 ± 0.08
		RCT‐Two‐stage model	0.93 ± 0.07
		Control‐One‐stage model	0.90 ± 0,18
		Control‐Two‐stage model	0.90 ± 0,18
Schmaranzer F.	2019	Single model	0.86 ± 0.03
Sezer A.	2023	Type 1	0.987
		Type 2a and 2b	0.968
		Type 2c and D	0.961
Shen H.	2021	Multifidus (left)	0.922 (0.026)
		Multifidus (right)	0.933 (0.026)
		Erector spinae (left)	0.911 (0.03)
		Erector spinae (right)	0.905 (0.024)
		Psoas major (left)	0.936 (0.018)
		Psoas major (right)	0.942 (0.016)
		Disc	0.959 (0.013)
		Spinal canal	0.902 (0.036)
Wang D.	2022	Local dataset‐Proximal femur	0.916
		Local dataset‐Fragment	0.804
		Local dataset‐Distal femur	0.870
		Cross dataset‐Proximal femur	0.871
		Cross dataset‐Fragment	0.697
		Cross dataset‐Distal femur	0.887
		Human‐computer competition‐	0.951
		Proximal femur	
		Human‐computer competition‐	0.876
		Fragment	
		Human‐computer competition‐	0.907
		Distal femur	
Wong V.	2023	3D original data	0.660
		3D augmented data	0.800
		2D axial‐original data	0.790
		2D axial‐augmented data	0.800
		2D coronal‐original data	0.800
		2D coronal‐augmented data	0.810
		2D sagittal‐original data	0.780
		2D augmented data	0.800
Wu S.	2023	Single model	0.915
Yang L.	2022	Single model	0.905
Yang M.	2022	OAI/R1	0.519
		APM/R1	0.018
		MIX/R1	0.741
		TL/R1	0.819
		TL/R2	0.785
		R1/R2	0.782
		TL/R1 (1.5 T)	0.797
		TL/R1 (3 T)	0.832
		Philips (1.5 T)	0.774
Zang Q.	2023	Single model	0.930
Zeng G. Degonda C.	2021	Acetabular Models	0.970 ± 0.02
		Femoral Models	0.980 ± 0.01
Zeng G. Schmaranzer F.	2021	Acetabular models	0.970 ± 0.02
		Femoral Models	0.980 ± 0.01
Zheng H.	2022	Vertebral body	0.959
		IVD	0.931
		Lumbar spine	0.934

Abbreviations: AI, artificial Intelligence; DSC, dice similarity coefficient; IVD, intervertebral disk.

**Table 3:** Accuracy.

First author	Year	Models	Accuracy
Ahn G.	2022	Single model	0.46
Chaudhari A.S.	2020	DeepResolve	0.90
		TCI	0.86
Hwang D.	2022	Single model	0.945
Jeon U.	2021	Medial meniscus	0.985
		Lateral meniscus	0.985
Kashua S.	2023	Single model	0.895
Kim H.	2023	Model 1	0.978
		Model 2	0.989
		Model 3	0.986
Kwolek K	2023	Single model	0.959
Lee S.	2024	Radius: AP X‐ray	0.998
		Radius: lateral X‐ray	0.997
		Ulna: AP X‐ray	0.998
Li X.	2023	Single model	0.977 ± 0.303
Luan K.	2020	Test set 1	0.998
		Test set 2	0.998
Schacky C.	2021	Single model	0.802
Wang G.	2020	AlexNet	0.940
		VGG16	0.880
		Inception3	0.880
		ResNet	0.900
Yang L.	2022	Single model	0.894
Zang Q.	2023	Single model	0.900
Zhang R.	2023	Using angle correction	0.730
		Without angle correction	0.650

First author	Year	Models	DSC
Alsinan A.	2020	Single model	0.67
Burton W.	2020	Single model	0.989
Chen T.	2022	VBS and IVD	0.918
El‐Hariri H.	2021	Single model	0.85
Flannery S.W.	2021	Single model	0.84
Flannery S.W.	2022	Single model	0.80
Hess H.	2023	Single model	0.91
Jeon U.	2021	Medial meniscus	0.851 ± 0.127
		Lateral meniscus	0.843 ± 0.126
Kim H.	2022	2D	0.810 ± 0.063
		3D	0.830 ± 0.073
Kim HS.	2024	Single model	0.84
Kim S	2018	Single model	0.894
Kim‐Wang S.Y.	2023	Validation set	0.985
		Testing set	0.984
Kok J.	2023	Healthy	0.95
		Scoliotic spine	0.93
Kokita S	2020	Average	0.955
Kulsen C.P.S	2023	Average	0.967 ± 0.040
Kuok C.P	2020	Chuang's dataset‐Tendon	0.93
		Chuang's dataset‐Synovial sheath	0.91
		MB dataset‐Tendon	0.92 ± 0.04
		MB dataset‐Synovial sheath	0.95 ± 0.03
Lee K.	2021	Single model	0.736
Lee SH.	2023	Single model	0.943
Li X.	2023	Single model	0.933 ± 0.955
Luan K.	2020	Test set 1	0.93
		Test set 2	0.912
Marsilio L.	2023	Single model	0,991
Masse‐Gignac N.	2023	Single model	0,953
Medina G	2021	Internal test data set	0.994
		External test data set	0.989
Mu X.	2021	Alex Net	0.910 ± 0.02
		VGG16	0.790 ± 0.02
		Inception 3	0.820 ± 0.02
		ResNet	0.800 ± 0.02
Nishiyama D.	2021	Single model	0.748
Olmez E.	2020	Single model	0.888 ± 0.075
Riem L.	2023	RCT‐One‐stage model	0.93 ± 0.08
		RCT‐Two‐stage model	0.93 ± 0.07
		Control‐One‐stage model	0.90 ± 0,18
		Control‐Two‐stage model	0.90 ± 0,18
Schmaranzer F.	2019	Single model	0.86 ± 0.03
Sezer A.	2023	Type 1	0.987
		Type 2a and 2b	0.968
		Type 2c and D	0.961
Shen H.	2021	Multifidus (left)	0.922 (0.026)
		Multifidus (right)	0.933 (0.026)
		Erector spinae (left)	0.911 (0.03)
		Erector spinae (right)	0.905 (0.024)
		Psoas major (left)	0.936 (0.018)
		Psoas major (right)	0.942 (0.016)
		Disc	0.959 (0.013)
		Spinal canal	0.902 (0.036)
Wang D.	2022	Local data set‐Proximal femur	0.916
		Local data set‐Fragment	0.804
		Local data set‐Distal femur	0.870
		Cross data set‐Proximal femur	0.871
		Cross data set‐Fragment	0.697
		Cross data set‐Distal femur	0.887
		Human‐computer competition‐	0.951
		Proximal femur	
		Human‐computer competition‐	0.876
		Fragment	
		Human‐computer competition‐	0.907
		Distal femur	
Wong V.	2023	3D original data	0.660
		3D augmented data	0.800
		2D axial‐original data	0.790
		2D axial‐augmented data	0.800
		2D coronal‐original data	0.800
		2D coronal‐augmented data	0.810
		2D sagittal‐original data	0.780
		2D augmented data	0.800
Wu S.	2023	Single model	0.915
Yang L.	2022	Single model	0.905
Yang M.	2022	OAI/R1	0.519
		APM/R1	0.018
		MIX/R1	0.741
		TL/R1	0.819
		TL/R2	0.785
		R1/R2	0.782
		TL/R1 (1.5 T)	0.797
		TL/R1 (3 T)	0.832
		Philips (1.5 T)	0.774
Zang Q.	2023	Single model	0.930
Zeng G. Degonda C.	2021	Acetabular Models	0.970 ± 0.02
		Femoral Models	0.980 ± 0.01
Zeng G. Schmaranzer F.	2021	Acetabular models	0.970 ± 0.02
		Femoral Models	0.980 ± 0.01
Zheng H.	2022	Vertebral body	0.959
		IVD	0.931
		Lumbar spine	0.934

Abbreviations: DSC, dice similarity coefficient; IVD, intervertebral disk; VBS, vertebral bone structures.

**Table 4:** Sensitivity.

First author	Year	Models	Sensitivity
Chaudhari A.S.	2020	DeepResolve	0.80
		TCI	0.63
Flannery S.W.	2021	Single model	0.85
Flannery S.W.	2022	Single model	0.82
Hess H.	2023	Single model	1.0
Hwang D.	2022	Single model	0.970
Jeon U.	2021	Medial meniscus	0.867 ± 0.489
		Lateral meniscus	0.856 ± 0.593
Kashua S.	2023	Single model	0.905
Lee S.	2024	Radius: AP X‐ray	0.979
		Radius: lateral X‐ray	0.955
		Ulna: AP X‐ray	0.965
Lee SH.	2023	Single model	0.971
Li X.	2023	Single model	0.911 ± 0.147
Mu X.	2021	Alex Net	0.950 ± 0.02
		VGG16	0.830 ± 0.02
		Inception 3	0.870 ± 0.02
		ResNet	0.830 ± 0.02
Olmez E.	2020	Single model	0.947 ± 0.052
Schacky C.	2021	Single model	0.629
Wang D.	2022	Local dataset‐Proximal femur	0.923
		Local dataset‐Fragment	0.786
		Local dataset‐Distal femur	0.862
		Cross dataset‐Proximal femur	0.853
		Cross dataset‐Fragment	0.764
		Cross dataset‐Distal femur	0.865
		Human‐computer competition‐	0.908
		Proximal femur	
		Human‐computer competition‐	0.824
		Fragment	
		Human‐computer competition‐	0.831
		Distal femur	
Wu S.	2023	Single model	0.897

First author	Year	Models	Accuracy
Ahn G.	2022	Single model	0.46
Chaudhari A.S.	2020	DeepResolve	0.90
		TCI	0.86
Hwang D.	2022	Single model	0.945
Jeon U.	2021	Medial meniscus	0.985
		Lateral meniscus	0.985
Kashua S.	2023	Single model	0.895
Kim H.	2023	Model 1	0.978
		Model 2	0.989
		Model 3	0.986
Kwolek K	2023	Single model	0.959
Lee S.	2024	Radius: AP X‐ray	0.998
		Radius: lateral X‐ray	0.997
		Ulna: AP X‐ray	0.998
Li X.	2023	Single model	0.977 ± 0.303
Luan K.	2020	Test set 1	0.998
		Test set 2	0.998
Schacky C.	2021	Single model	0.802
Wang G.	2020	AlexNet	0.940
		VGG16	0.880
		Inception3	0.880
		ResNet	0.900
Yang L.	2022	Single model	0.894
Zang Q.	2023	Single model	0.900
Zhang R.	2023	Using angle correction	0.730
		Without angle correction	0.650

First author	Year	Models	DSC
Alsinan A.	2020	Single model	0.67
Burton W.	2020	Single model	0.989
Chen T.	2022	VBS and IVD	0.918
El‐Hariri H.	2021	Single model	0.85
Flannery S.W.	2021	Single model	0.84
Flannery S.W.	2022	Single model	0.80
Hess H.	2023	Single model	0.91
Jeon U.	2021	Medial meniscus	0.851 ± 0.127
		Lateral meniscus	0.843 ± 0.126
Kim H.	2022	2D	0.810 ± 0.063
		3D	0.830 ± 0.073
Kim HS.	2024	Single model	0.84
Kim S	2018	Single model	0.894
Kim‐Wang S.Y.	2023	Validation set	0.985
		Testing set	0.984
Kok J.	2023	Healthy	0.95
		Scoliotic spine	0.93
Kokita S	2020	Average	0.955
Kulsen C.P.S	2023	Average	0.967 ± 0.040
Kuok C.P	2020	Chuang's dataset‐Tendon	0.93
		Chuang's dataset‐Synovial sheath	0.91
		MB dataset‐Tendon	0.92 ± 0.04
		MB dataset‐Synovial sheath	0.95 ± 0.03
Lee K.	2021	Single model	0.736
Lee SH.	2023	Single model	0.943
Li X.	2023	Single model	0.933 ± 0.955
Luan K.	2020	Test set 1	0.93
		Test set 2	0.912
Marsilio L.	2023	Single model	0,991
Masse‐Gignac N.	2023	Single model	0,953
Medina G	2021	Internal test dataset	0.994
		External test dataset	0.989
Mu X.	2021	Alex Net	0.910 ± 0.02
		VGG16	0.790 ± 0.02
		Inception 3	0.820 ± 0.02
		ResNet	0.800 ± 0.02
Nishiyama D.	2021	Single model	0.748
Olmez E.	2020	Single model	0.888 ± 0.075
Riem L.	2023	RCT‐One‐stage model	0.93 ± 0.08
		RCT‐Two‐stage model	0.93 ± 0.07
		Control‐One‐stage model	0.90 ± 0,18
		Control‐Two‐stage model	0.90 ± 0,18
Schmaranzer F.	2019	Single model	0.86 ± 0.03
Sezer A.	2023	Type 1	0.987
		Type 2a and 2b	0.968
		Type 2c and D	0.961
Shen H.	2021	Multifidus (left)	0.922 (0.026)
		Multifidus (right)	0.933 (0.026)
		Erector spinae (left)	0.911 (0.03)
		Erector spinae (right)	0.905 (0.024)
		Psoas major (left)	0.936 (0.018)
		Psoas major (right)	0.942 (0.016)
		Disc	0.959 (0.013)
		Spinal canal	0.902 (0.036)
Wang D.	2022	Local dataset‐Proximal femur	0.916
		Local dataset‐Fragment	0.804
		Local dataset‐Distal femur	0.870
		Cross dataset‐Proximal femur	0.871
		Cross dataset‐Fragment	0.697
		Cross dataset‐Distal femur	0.887
		Human‐computer competition‐	0.951
		Proximal femur	
		Human‐computer competition‐	0.876
		Fragment	
		Human‐computer competition‐	0.907
		Distal femur	
Wong V.	2023	3D original data	0.660
		3D augmented data	0.800
		2D axial‐original data	0.790
		2D axial‐augmented data	0.800
		2D coronal‐original data	0.800
		2D coronal‐augmented data	0.810
		2D sagittal‐original data	0.780
		2D augmented data	0.800
Wu S.	2023	Single model	0.915
Yang L.	2022	Single model	0.905
Yang M.	2022	OAI/R1	0.519
		APM/R1	0.018
		MIX/R1	0.741
		TL/R1	0.819
		TL/R2	0.785
		R1/R2	0.782
		TL/R1 (1.5 T)	0.797
		TL/R1 (3 T)	0.832
		Philips (1.5 T)	0.774
Zang Q.	2023	Single model	0.930
Zeng G. Degonda C.	2021	Acetabular Models	0.970 ± 0.02
		Femoral Models	0.980 ± 0.01
Zeng G. Schmaranzer F.	2021	Acetabular models	0.970 ± 0.02
		Femoral Models	0.980 ± 0.01
Zheng H.	2022	Vertebral body	0.959
		IVD	0.931
		Lumbar spine	0.934

Abbreviations: DSC, dice similarity coefficient; IVD, intervertebral disk; VBS, vertebral bone structures.

**Table 5:** Precision.

First author	Year	Models	Precision
Chen T.	2022	VBS and IVD	0.922
Flannery S.W.	2021	Single model	0.82
Flannery S.W.	2022	Single model	0.79
Kashua S.	2023	Single model	0.894
Lee K.	2021	Single model	0.604
Lee SH.	2023	Single model	0.849
Luan K.	2020	Test set 1	0.951
		Test set 2	0.904
Marsilio L.	2023	Single model	0.997
Medina G	2021	Internal test dataset	0.994
		External test dataset	0.985
Sàeenz‐Gamboa J.	2023	U1 TH	0.960
		UMD TH	0.960
		E 12 NAD‐TH	0.960
Walle M.	2022	Single model	0.916 ± 0.019
Wang D.	2022	Local dataset‐Proximal femur	0.996
		Local dataset‐Fragment	0.762
		Local dataset‐Distal femur	0.877
		Cross dataset‐Proximal femur	0.914
		Cross dataset‐Fragment	0.740
		Cross dataset‐Distal femur	0.915
		Human‐computer competition‐	0.916
		Proximal femur	
		Human‐computer competition‐	0.853
		Fragment	
		Human‐computer competition‐	0.888
		Distal femur	
Zeng G. Degonda C.	2021	Acetabular Models	0.960 ± 0.03
		Femoral Models	0.980 ± 0.02
Zeng G. Schmaranzer F.	2021	Acetabular models	0.960 ± 0.03
		Femoral Models	0.980 ± 0.02

Abbreviations: IVD, intervertebral disk; VBS, vertebral bone structures.

**Table 6:** Specificity.

First author	Year	Models	Specificity
Chaudhari A.S.	2020	DeepResolve	0.93
		TCI	0.91
Hess H.	2023	Single model	0.94
Hwang D.	2022	Single model	0.926
Jeon U.	2021	Medial meniscus	0.991 ± 0.42
		Lateral meniscus	0.991 ± 0.20
Lee S.	2024	Radius: AP X‐ray	0.999
		Radius: lateral X‐ray	0.998
		Ulna: AP X‐ray	0.999
Lee SH.	2023	Single model	0.95
Luan K.	2020	Test set 1	0.994 ± 0.52
		Test set 2	0.999
Marsilio L.	2023	Single model	0.998
Schmaranzer F.	2019	Single model	0.882

**Table 7:** F1.

First author	Year	Models	F1
Alsinan A.	2019	Single model	95%
Jecklin S.	2022	Single model	0.88
Kashua S.	2023	Single model	0.894
Lee SH.	2023	Single model	0.905

**Table 8:** AUC.

First author	Year	Models	AUROC
Hwang D.	2022	Single model	0.988
Kashua S.	2023	Single model	0.931
Zang Q.	2023	Single model	0.940

Abbreviation: AUC, area under the curve; ROC, receiver operating characteristic.

**Table 9:** Clinical applications.

Model	Type	Description	Primary applications
AI	General Term	General term for artificial intelligence	Various applications across industries
GAN	Generative Model	Generative Adversarial Network, consists of a generator and a discriminator	Image generation, data augmentation, unsupervised learning
CNN	Convolutional Network	Convolutional Neural Network, excels in image and spatial data processing	Image classification, object detection, medical imaging
3D U‐Net	Convolutional Network	Extension of U‐Net for 3D data	3D medical image segmentation
3D CNN	Convolutional Network	CNN adapted for 3D data	Video analysis, 3D medical imaging
2D U‐Net	Convolutional Network	U‐Net architecture for 2D image data	2D medical image segmentation, satellite image analysis
nnU‐Net	Convolutional Network	No new net, an automatically adapting U‐Net framework	Medical image segmentation, adaptable to various data sets
U‐Net	Convolutional Network	CNN architecture designed for biomedical image segmentation	Biomedical image segmentation, image restoration
XGBoost	Ensemble Method	Gradient boosting framework	Structured/tabular data analysis, regression, classification
X23D algorithm	Algorithm	Specific algorithm (details not provided)	Depends on specific use case
2D CNN	Convolutional Network	CNN for 2D data	Image recognition, classification, segmentation
DeepLab v3+	Convolutional Network	Advanced semantic image segmentation model	Semantic segmentation.
UNETR	Transformer‐based	U‐Net architecture using transformers	Medical image segmentation.
BSU‐Net	Convolutional Network	Specific U‐Net variant (details not provided)	Depends on specific use case
DenseVNet	Convolutional Network	Dense connectivity V‐Net for volumetric data	Medical image analysis
D2FC‐DN	Convolutional Network	Specific CNN variant (details not provided)	Depends on specific use case
Yolo	Object Detection	You Only Look Once, real‐time object detection system	Real‐time object detection
SMART‐CA	Specific Algorithm	Specific algorithm (details not provided)	Depends on specific use case
MsgeCNN	Convolutional Network	Specific CNN variant (details not provided)	Depends on specific use case
BoneNet	Convolutional Network	Specific CNN for bone analysis (details not provided)	Medical imaging (bone analysis)
CEL‐Unet	Convolutional Network	Specific U‐Net variant (details not provided)	Depends on specific use case
Alex Net	Convolutional Network	Early deep CNN model that won ImageNet 2012	Image classification
R‐CNN	Object Detection	Region‐based CNN for object detection.	Object detection, image segmentation
DL	General Term	Deep Learning, subset of machine learning using neural networks	Various applications across industries
3‐D dGEMRIC	MRI Analysis	3D delayed gadolinium‐enhanced MRI of cartilage	Cartilage imaging and analysis
Mask R‐CNN	Object Detection	Extension of R‐CNN for instance segmentation	Instance segmentation, object detection.
Spine Explorer (Tulong)	Specific System	Specific system for spinal analysis (details not provided)	Spinal image analysis.
V‐Net	Convolutional Network	3D convolutional network for volumetric medical image segmentation	Volumetric medical image segmentation
PCNN	Convolutional Network	Specific CNN variant (details not provided)	Depends on specific use case
FCN	Convolutional Network	Fully Convolutional Network for semantic segmentation	Semantic segmentation
FCNN‐4s,CRF	Convolutional Network	Specific CNN and Conditional Random Field variant (details not provided)	Depends on specific use case
DenseUnet	Convolutional Network	U‐Net with dense connections	Medical image segmentation
cGAN	Generative Model	Conditional Generative Adversarial Network, generates data conditioned on input	Image‐to‐image translation
3D LP‐U‐net	Convolutional Network	Specific U‐Net variant for 3D data (details not provided)	Depends on specific use case
VDVM	Specific Algorithm	Specific algorithm (details not provided)	Depends on specific use case
BianqueNet	Specific Network	Specific network (details not provided)	Depends on specific use case

Abbreviations: 3D dGEMRIC, 3D Delayed gadolinium‐enhanced MRI of cartilage; 3D‐UNet, 3D U‐shaped architecture network; AI, artificial Intelligence; cGAN, conditional generative adversarial network; CNN, convolutional neural network; CT, computed tomography; DR, digital radiography; FCN, full convolutional neural networks; FCNN‐4s, fully convolutional neural network; MRI, magnetic resonance imaging; nnU‐Net, not new network; PCNN, pulse coupled neural network; SMART‐CA, Segmentation Model Adopting a pRe‐trained Classification Architecture; VDVM, vertebral segment matching.

### Data items

Studies were screened based on the imaging techniques and ML models under investigation.

This documentation proved crucial for comparing the efficacy of ML models across multimodal imaging techniques representing diverse joints/body regions. An exhaustive collection of outcome metrics was undertaken. The model's discriminative capacity between conditions was gauged through the AUC. The DSC is employed as an essential measure to quantitatively assess the accuracy and overlap of image segmentation algorithms, crucial for delineating anatomical structures in clinical imaging. To assess the overall correctness of the models' predictions, accuracy was employed as a key indicator of their reliability in clinical settings. Equally important was the balance between precision and recall, encapsulated in the F1 Score; specificity further complemented this analysis by highlighting the models' aptitude in accurately identifying true negatives.

To ensure the robustness of this comparison and the relevance of the findings, outcome metrics were included in this analysis only if three or more studies reported them.

Results pertaining to each outcome domain were collected uniformly. This comprehensive approach ensured that this review encapsulated the full spectrum of available evidence regarding the efficacy of ML models in the enhancement of multimodal imaging techniques.

### Study risk of bias assessment

Two independent reviewers (G.N., M.G.P.), assessed the methodological quality of included studies, a third reviewer (A.L.) was consulted if discrepancies were not resolved by discussion. Two different tools, the Joanna Briggs Institute (JBI) Critical Appraisal tool [48], and the Risk Of Bias In Non‐randomized Studies‐of Interventions (ROBINS‐I) [14] tool were chosen for the methodological quality assessment of the included studies, for case‐series and case‐control studies respectively. The tools were used to assess the risk of bias as high, moderate, or low.

The JBI provides critical appraisal tools to assess the trustworthiness, relevance, and results of published papers. The instrument includes several key questions that help evaluate the quality and potential bias in research studies, contributing to robust evidence synthesis and informed decision‐making [48].

The ROBINS‐I tool defines a set of domains and provide ‘signaling questions’ designed to evaluate different biases within a study. Each domain can be scored as having a low, moderate, serious, or critical risk of bias, and an overall score can be assigned to each study [14].

## RESULTS

### Study selection

A total of 1348 records were found and screened for title and abstract (Figure 1). No duplicates were found. Seven hundred forty‐five were excluded, leaving 603 articles for full‐text screening. The full‐text screening of the selected articles verified if they met the inclusion criteria and excluded a total of 550 studies. Reasons for exclusion at full‐text screening included: 390 studies due to unrelated topic, 147 due to insufficient data, three due to use of deterministic algorithms, seven were systematic reviews, three were protocol studies. Fifty‐three articles were included in the systematic review and results presented in Tables 1, 2, 3, 4, 5, 6, 7, 8, 9 [1, 2, 3, 4, 5, 6, 7, 9, 10, 12, 13, 15, 16, 18, 20, 21, 22, 23, 24, 25, 26, 28, 29, 31, 33, 34, 35, 36, 39, 42, 43, 44, 45, 46, 47, 49, 50, 51, 52, 53, 54, 55, 56, 57, 58, 59, 60, 61, 62, 63, 64, 65, 67].

### Study characteristics

All included studies involved the application of ML models to real patient images, sourced from databases collected either retrospectively or prospectively with small to large sample sizes (8‐11.891.000); [20, 26] in total, 11.990.643 images were analysed in the studies object of this systematic review. This screening of integration of AI in clinical practice included data sets of X‐Ray, US, MRI and CT, the quality and segmentation ability of which were enhanced by ML models embracing Convolutional Neural Network (CNN), Generative Adversarial Network (GAN), U‐Net, eXtreme Gradient Boosting (XGBoost), X23D algorithm, DeepLab v3 + , Bilaterally Symmetric U‐Shaped Network (BSU‐Net), DenseVNet, Deeply supervised dilated fully convolutional DenseNet (D2FC‐DN), YOLO (You only look once), Segmentation Model Adopting a pRe‐trained Classification Architecture, (SMART‐CA), Bone‐Net, Alex Net, 3D Delayed gadolinium‐enhanced MRI of cartilage (3D dGEMRIC), Spine Explorer, V‐Net, Convolutional random field (CRF), Vertebral segment matching (VDVM) and BianqueNet (Table 2).

#### Improving image quality

Ahn et al. [1]: Utilized a GAN to generate realistic knee X‐ray images, achieving an accuracy of 0.46.

Chaudhari et al. [5]: Leveraged a CNN for knee joint analysis through 3D DESS MRI super‐resolution, improving image clarity with an accuracy of 0.90 (DeepResolve) and 0.86 (TCI).

Wang G. et al. [56]: Combined Pulse‐coupled neural network (PCNN) and fullCNN to improve MRI image quality of the humerus and shoulder joint, achieving accuracies of 0.94 (AlexNet), 0.88 (VGG16), 0.88 (Inception3), and 0.90 (ResNet).

The image quality enhancement models showed improved image realism and clarity, with accuracies ranging from 0.46 to 0.90.

#### Automating segmentation and measurement

Alsinan et al. [3]: Proposed a CNN‐based method for automated bone segmentation, achieving a Dice Similarity Coefficient (DSC) of 0.67.

Burton et al. [4]: Explored CNN‐based automatic segmentation of the knee joint from MRI with a DSC of 0.98.

Chen et al. [6]: Investigated the feasibility of automatically segmenting spinal structures, achieving a DSC of 0.91.

El‐Hariri et al. [7]: Validated a 3D CNN for segmenting DDH metrics in the hip joint on US with a DSC of 0.85.

Flannery et al. [10]: Deployed a 2D U‐Net for ACL segmentation with a DSC of 0.84, and in 2022 validated a DL model for ACL reconstruction with a DSC of 0.80.

Hwang et al. [13]: Predicted sarcopenia with a U‐Net and XGBoost model, achieving an accuracy of 0.94 and sensitivity of 0.97.

Kim et al. [21]: Automated segmentation of rotator cuff tears on T2 MRI images with DSC scores of 0.81 (2D) and 0.83 (3D).

Lee et al. [34]: Measured distal radius fractures with a U‐Net technique, achieving accuracies of 0.99 (AP X‐ray) and 0.99 (lateral X‐ray).

Riem et al. [47]: Validated a 3D U‐Net algorithm for RC muscle segmentation with DSC scores of 0.93 for one‐stage and two‐stage models.

The segmentation models achieved high performance metrics, with DSC ranging from 0.67 to 0.98 and accuracy scores up to 0.99.

#### Generating synthetic images

Alsinan et al. [3]: Introduced a GAN‐based architecture for bone shadow image segmentation, achieving DSC scores of 0.93 (Chuang's data set‐Tendon), 0.91 (Chuang's dataset‐Synovial sheath), 0.92 (MB data set‐Tendon), and 0.95 (MB data set‐Synovial sheath).

Yang et al. [60]: Built a cGANs model to automatically segment knee MRI scans, achieving DSC scores ranging from 0.51 to 0.83 depending on the dataset.

The generative models showed capability in creating synthetic images with DSC ranging from 0.51 to 0.95.

#### Developing predictive models

Jecklin et al. [15]: Introduced a DL‐based X23D algorithm for lumbar vertebrae shape estimation, achieving an F1 score of 0.88.

Jeon et al. [16]: Developed a two‐stage DCNN for knee MRI analysis, achieving DSC scores of 0.85 (medial meniscus) and 0.84 (lateral meniscus).

Kim et al. [20]: Applied a DeepLab v3+ model for tibia and fibula fracture segmentation, achieving DSC scores of 0.97 (Model 1), 0.98 (Model 2), and 0.98 (Model 3).

Li et al. [36]: Proposed a MsgeCNN‐based framework for femoral head necrosis diagnosis, achieving a DSC of 0.93 and an accuracy of 0.97.

The predictive models demonstrated high segmentation accuracy with DSC ranging from 0.84 to 0.98 and F1 scores up to 0.88.

#### Other specific applications

Marsilio et al. [42]: Introduced an AI‐based CEL‐Unet for 3D reconstruction in total knee arthroplasty planning, achieving a DSC of 0.99 and a precision of 0.99.

Nishiyama et al. [46]: Automated gluteus medius segmentation in hip osteoarthritis diagnosis, achieving a DSC of 0.74.

Schmaranzer et al. [49]: Implemented a 3‐D dGEMRIC technique for cartilage thickness analysis, achieving a DSC of 0.86.

Wong et al. [57]: Automated scapula segmentation with a CNN, achieving DSC scores ranging from 0.66 (3D original data) to 0.81 (2D augmented data).

Zhang et al. [63]: Developed a cartilage segmentation method with nnU‐Net, achieving a DSC of 0.93.

Zheng et al. [65]: Quantified intervertebral disc degeneration with BianqueNet, achieving DSC scores of 0.95 (vertebral body), 0.93 (IVD), and 0.93 (lumbar spine).

The specific application models achieved DSC ranging from 0.66 to 0.99 and precision scoredup to 0.99.

### Risk of bias assessment

**Figure 2:**
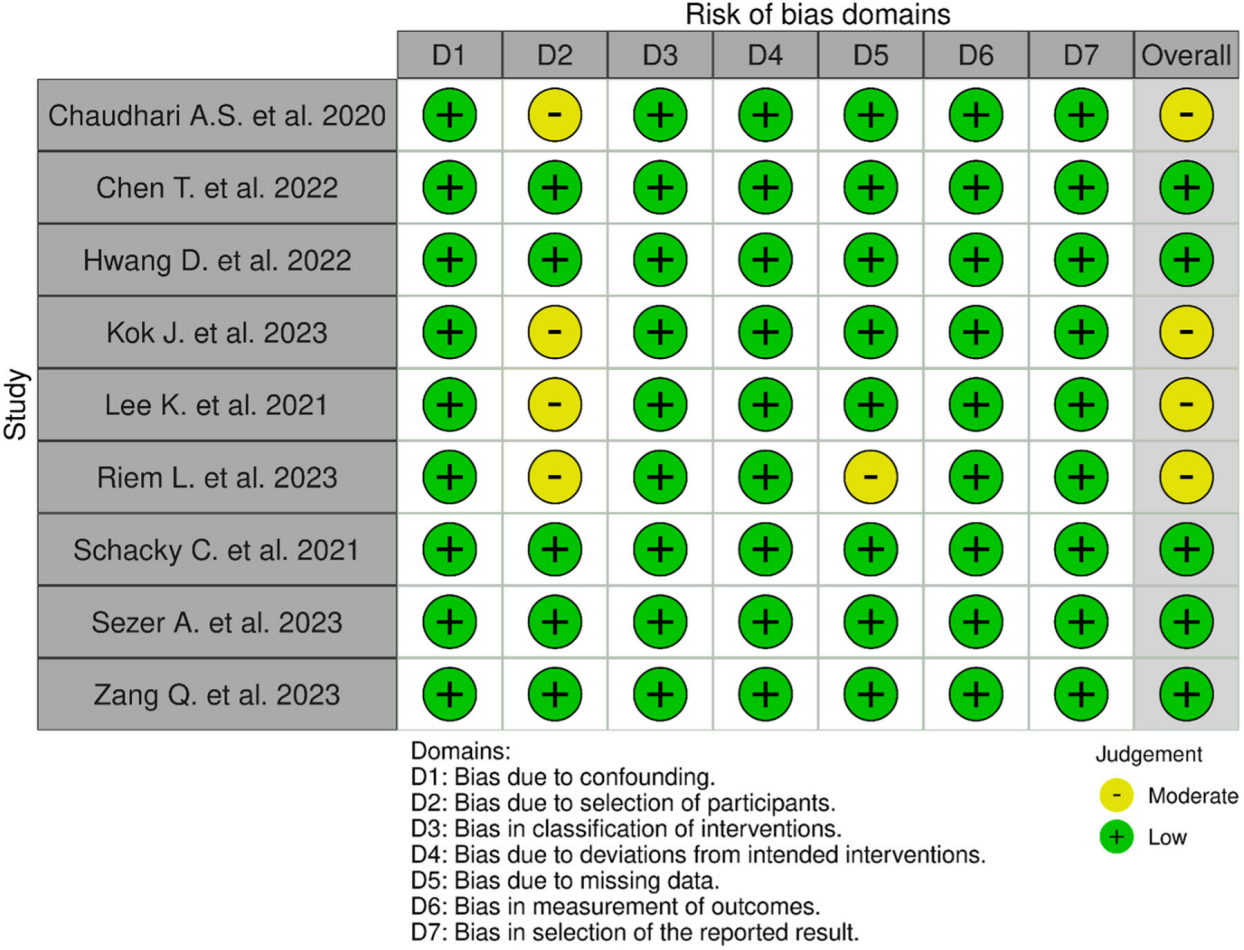
ROBINS‐I risk‐of‐bias tool for case‐control studies.

**Figure 3:**
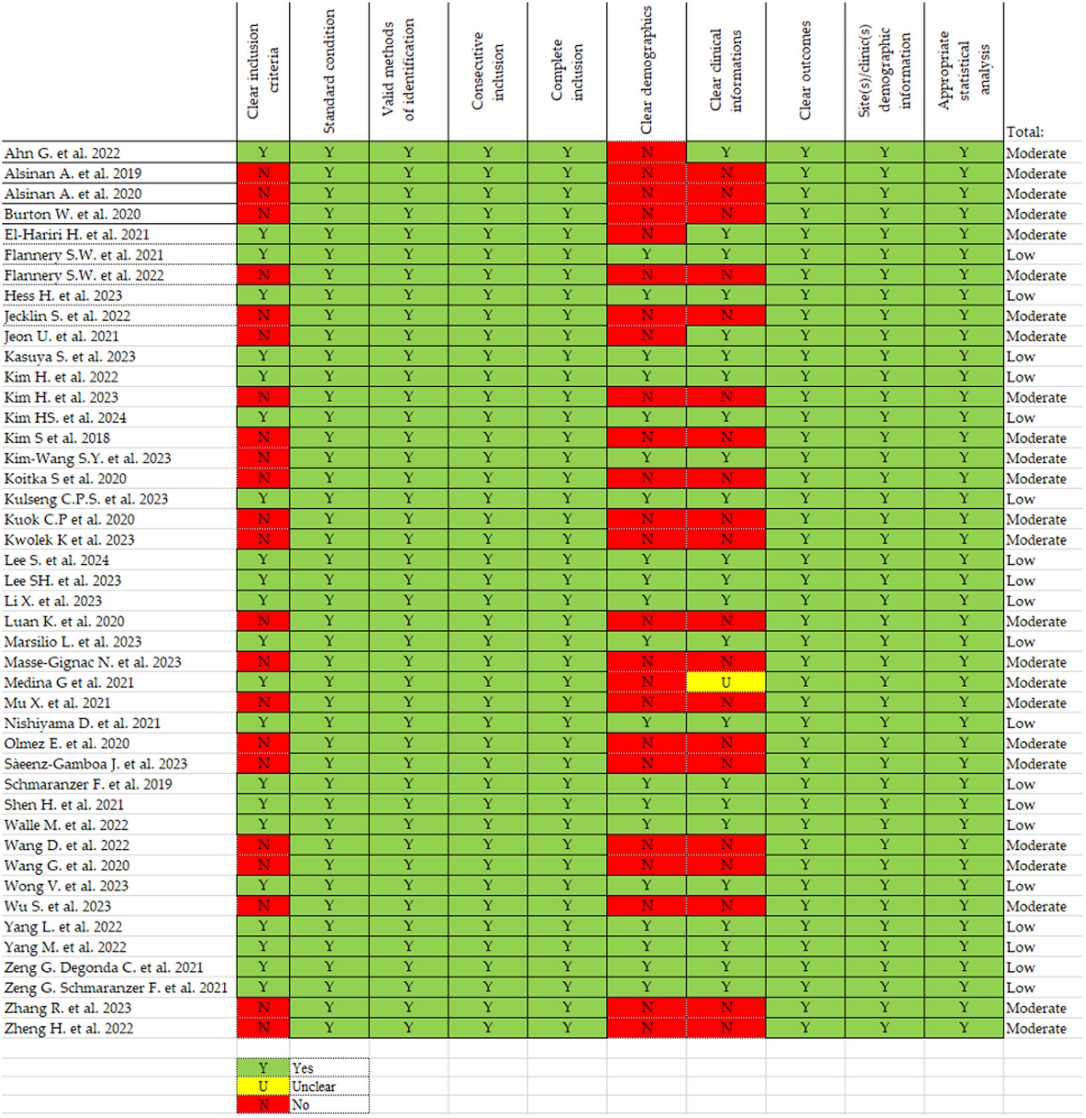
Risk of bias of included CSs evaluated using the Joanna Briggs Institute Critical Appraisal Tool for case‐series.

For case‐series, the following items were rated: clear inclusion criteria, clinical information, demographics and outcomes, standard condition, valid methods of identification, appropriate statistical analysis. For case‐control the items were: bias due to confounding, to selection of participants, to deviations from intended interventions, to missing data, bias in classification of interventions, in the measurement of outcomes and bias in the selection of the reported results (Figures 2 and 3). Studies were rated as having a high, low, or moderate risk of bias for each component of the tool. Almost all the studies obtained a low risk of bias concerning clear outcomes, highlighting the rigorous mathematical foundation inherent within the field of AI. This framework quantitatively assesses the output variable with marked scientific precision. The inclusion criteria of participants and their clinical information obtained a high risk of bias, due to the nature of the studies that did not focus on a follow‐up of the patients but more about the prediction capability of the ML models.

## DISCUSSION

This systematic review underscores the significant role of AI in advancing multimodal imaging in orthopaedics. AI technologies, particularly through DL techniques such as CNNs and GANs, handle complex image segmentation tasks and provide detailed visualizations, which are vital for accurate diagnostics and effective treatment planning. Our analysis showed high performance metrics across various models and imaging tasks, including DSC, accuracy, sensitivity and precision [11, 40, 41, 66].

Federer et al. showed the use of AI in clinical decision‐making by using large digital databases. Their study found better diagnostic accuracy for spinal pathologies, hip fractures and knee injuries using MRI and CT scans [8].

AI models like GANs and CNNs have been particularly successful in improving image realism and clarity, essential for better diagnostics. Enhanced image quality facilitates more accurate diagnoses and reduces the likelihood of misinterpretations, which is crucial in clinical settings. Future research should focus on refining these models for broader clinical applications and standardizing the evaluation metrics used. This finding aligns with recent literature that demonstrates AI's ability to optimize image quality in orthopaedic diagnostics, leading to more reliable clinical outcomes [8].

In terms of automating segmentation and measurement, AI models like U‐Net and its variants demonstrated high accuracy. Automated segmentation reduces the time required for manual analysis and increases consistency and reproducibility in measurements, crucial for preoperative planning and postoperative assessments. Future work should aim to integrate these automated systems into routine clinical workflows and validate their performance across larger, more diverse populations.

This coincides with previous evidences: Kurmis and Ianunzio [30] demonstrated that models like U‐Net and its variants can achieve high segmentation accuracy, as evidenced by their application in knee joint and rotator cuff segmentation.

GANs were effective in generating synthetic images of the knee joint, which can augment training data sets and improve the robustness of AI models. Generating synthetic images is a growing area in AI research and could also be used as an additional feature to improve the accuracy of ultrasound (US)‐based registration methods. Further studies should explore the integration of these synthetic data sets into model training and their impact on real‐world diagnostic performance.

Predictive models also showed high accuracy in diagnosing conditions and planning treatments. These models can assist clinicians in making more accurate and timely decisions, potentially improving patient outcomes. This area has significant potential for enhancing clinical decision‐making. Future research should focus on validating these models in prospective, randomized controlled trials to establish their clinical efficacy.

Despite promising results, the studies exhibit great variability in methodological quality, affecting the generalizability of the findings. The predominant reliance on retrospective cohorts and the exclusion of randomized controlled trials limits the robustness of the conclusions. The sample sizes vary significantly among studies, with some based on small, potentially non‐representative patient populations, which may skew the efficacy and accuracy results of the AI applications. Additionally, the diversity in AI models, imaging techniques, anatomical complexities, and varying statistical measures used across studies contributes to extensive heterogeneity in outcomes. This makes it difficult to directly compare results and determine which AI features or models are most effective for specific orthopedic applications.

The extensive heterogeneity indicates the necessity for individualized model training and validation protocols tailored to specific imaging and diagnostic requirements to ensure the highest efficacy and utility in clinical settings. The integration of AI into orthopedic imaging promises substantial impacts on patient management and treatment outcomes. These findings underline the need for the development of guidelines to standardize AI implementation in medical imaging, ensuring these technologies are safely and effectively integrated into healthcare systems. Future research should address the current study's limitations by incorporating larger, randomized controlled trials to provide stronger evidence of AI's benefits.

For clinical applications, the review highlights the importance of integrating AI tools into orthopaedic practices, accompanied by sufficient training for clinicians to effectively interpret and utilize AI‐enhanced data. Furthermore, to deepen our comprehension of ML in medical research, a shift from patient‐centred to AI‐centred research is strongly recommended. This shift demands alternative methods for categorizing studies since traditional evidence levels like 'case‐control' become less applicable. Using the same patient dataset, various ML models can be tested, each with different parameters, emphasizing the need for a unique evaluation framework specific to ML in medicine.

## AUTHOR CONTRIBUTIONS

*Conceptualization*: Umile Giuseppe Longo. *Methodology*: Matteo Giuseppe Pisani, Guido Nicodemi, and Alberto Lalli. *Software, validation*: Umile Giuseppe Longo. *Formal analysis*: Alberto Lalli. *Investigation*: Guido Nicodemi and Matteo Giuseppe Pisani. *Writing—original draft preparation*: Guido Nicodemi and Matteo Giuseppe Pisani. *Writing—review and editing*: Alberto Lalli and Jacob F. Oeding. *Supervision*: Umile Giuseppe Longo. *Project administration*: Umile Giuseppe Longo. All authors have read and agreed to the published version of the manuscript.

## CONFLICT OF INTEREST STATEMENT

K.S is a member of the board of directors at Getinge AB (publ) and medtech advisor to Carl Bennet AB.

## ETHICS STATEMENT

The authors have nothing to report.

## Data Availability

All data generated or analysed during this study are included in this published article and its supplementary information files.
